# Symptom Experience Among Family Caregivers: Symptom Cluster Analysis

**DOI:** 10.1093/geroni/igaa057.497

**Published:** 2020-12-16

**Authors:** Yu-Ping Chang, Young Seo, Tania Von Visger

**Affiliations:** University at Buffalo, Buffalo, New York, United States

## Abstract

Family caregivers of older adults experience physical and psychological symptoms such as fatigue, insomnia, depression, or anxiety which may negatively impact their quality of life. Although those symptoms often co-occur, there is no prior research on symptom clusters identification among family caregivers. Symptom cluster analysis in chronic disease populations demonstrates usefulness in clinical management. An in-depth understanding of the pattern of symptom experience can guide intervention development to improve caregiving experience, coping skills, and well-being. This study aimed to identify symptom clusters as experienced by family caregivers in the US. We analyzed responses of 2,652 unpaid caregivers from the National Study of Caregiving (NSOC) III (2017) survey to identify underlying symptom clusters. The NSOC is a nationally representative survey of family caregivers to older adults with limited functions in daily activities. We used 17 binary items measuring the physiological and psychological symptoms of caregivers. We conducted a hierarchical cluster analysis with Ward linkage method to identify the symptom clusters. Three symptom clusters were identified based on the optimum silhouette width as follows: (1) Cluster 1 (lack of cheerfulness, peacefulness, and full of life, breathing problems, and sleep interrupted); Cluster 2 (feeling lonely, down, bored, nervous, worrisome, upset, and having little interest); and (3) Cluster 3 (limited strengths in arms and legs, having low energy, pain, and troubles falling back asleep). Our findings indicate that physical and psychological symptoms are highly preventable in family caregivers. Future research on symptoms management strategies can focus on targeting multiple symptoms based on their co-occurence.

